# Beyond *Access Microbiology*: implementing an open research platform for the Microbiology Society

**DOI:** 10.1099/acmi.0.000369

**Published:** 2022-07-14

**Authors:** Helina Marshall, Georgios Efthimiou

**Affiliations:** ^1^​ Queen’s University Belfast, Belfast, UK; ^2^​ University of Hull, Hull, UK

## What is Open Science?

In an attempt to make scientific research and its dissemination accessible to all levels of society, the Open Science movement has emerged. Its aim is to make knowledge more transparent, accessible, and reusable, so that it is easily shared and developed through professional or social networks. It involves promoting open research publication, campaigning for open access and pursuing a wider dissemination and engagement in science through transparently providing access to all the component aspects of scientific enquiry. These developments make it easier to publish data and code, access manuscripts and communicate scientific knowledge.

## What is an open research platform?

An open research platform is a combination of a preprint server and a scientific journal, aiming to improve the rigour, reproducibility and transparency of the academic record. The uploaded preprint will be visible on the online platform and after going through the review process it may be published (Version of Record). The readers of the article will be able to navigate through its history, reviews, and previous versions – watching the peer review process in action, and seeing how the article is improved before moving from a posted preprint to a published article. This will increase the transparency of the review process and help the readers assess the robustness of the manuscript’s content. Our open research platform will accept a variety of manuscript types such as Research Articles, Short Communications, Methods, Study Protocols, Data Notes, and Case Reports. It will also be considering new article types in the coming months and years, in response to the needs of our community.

## How will *Access Microbiology* be different?

There are many very exciting innovations in our *Access Microbiology* open research platform! More specifically, we are aiming to include a series of manuscript review tools on the platform. Before posting their manuscript as preprint, authors will have the chance to use these tools to improve their article. For example, three pieces of software will check that the article complies with our editorial or ethical policies, examine whether the genome sequences that are included are correctly linked and accessible, and detect areas of the text where language has been accidentally replicated from other pieces of published research. We have spent a number of months working with the developers of these tools, testing them and presenting them during a series of focus groups to determine their usefulness for reviewers, authors, Editors and readers. These reports will be accessible for readers alongside the peer review reports and the Editor’s decision comments.

## Why should you choose to publish on our open research platform?

First of all, for the authors, publishing in *Access Microbiology* can improve the discoverability, reuse and citability of their work by making the data open and easy to access by clearly referring to the data deposited in their article. Authors can communicate their research rapidly upon conclusion of their experiments, receiving a citable DOI while the peer review process continues. Furthermore, for the research community, preprints posted on the platform can facilitate future collaborations and can improve the speed at which important research can be conducted and disseminated. Finally, in the fields of environmental research and healthcare, the real-world impact of research is enhanced by its rapid and effective dissemination, which can then affect policy decisions and practical response plans, and allows these researchers to ensure the precedence of their discoveries.

**Editors-in-Chief for** ***Access Microbiology***

